# Language barriers and mental health problems of preschool children born very preterm in Germany

**DOI:** 10.1111/dmcn.16132

**Published:** 2024-10-21

**Authors:** Julia Jaekel, Nils Jaekel, Christoph Härtel, Wolfgang Göpel, Egbert Herting, Ursula Felderhoff‐Müser, Britta M. Huening, Juliane Spiegler

**Affiliations:** ^1^ Unit of Psychology, Faculty of Education and Psychology University of Oulu Oulu Finland; ^2^ Department of Paediatrics I, Neonatology, Paediatric Intensive Care, Paediatric Neurology University Hospital Essen, University of Duisburg‐Essen Essen Germany; ^3^ Department of Psychology University of Copenhagen Copenhagen Denmark; ^4^ Department of Psychology University of Warwick Warwick UK; ^5^ Department of English, German and Romance Studies, Faculty of Humanities University of Copenhagen Copenhagen Denmark; ^6^ Department of Paediatrics University Hospital Würzburg Würzburg Germany; ^7^ Department of Paediatrics University Hospital Lübeck Lübeck Germany; ^8^ Center of Translational Neuro‐ and Behavioural Sciences, C‐TNBS, Faculty of Medicine University of Duisburg‐Essen Essen Germany

## Abstract

**Aim:**

We assessed whether behavioural and emotional problems of 5‐ to 6‐year‐old preschool children born very preterm (<32 weeks' gestation) are associated with an immigrant background and linguistic distance of their first language to the host country's official language, German.

**Method:**

This is an observational longitudinal cohort study. Data are from the national multicentre German Neonatal Network cohort, including all very preterm births from 2009 onwards. A total of 3220 (*n* = 1570 female) children were followed up at preschool age; 629 (*n* = 324 female) of these had an immigrant background. Behavioural and emotional problems were assessed using the parent‐reported Strengths and Difficulties Questionnaire (SDQ).

**Results:**

Mixed‐effects models showed that immigrant status alone was not associated with children's behavioural and emotional problems. However, a higher linguistic distance of the children's first language to German was associated with higher SDQ total problem scores (coefficient = 0.008, 95% confidence interval 0.002, 0.015), after adjusting for known confounders.

**Interpretation:**

Language barriers in the form of linguistic distance between the first language of children born very preterm and countries' official languages are associated with increased risk for behavioural and emotional problems. More research is needed on how language barriers affect long‐term developmental outcomes of immigrant children born very preterm.

## Graphical abstract

Immigrant children born very preterm face systemic inequalities such as language barriers. Language barriers can be operationalized as a continuous linguistic distance score. Linguistic distance is associated with mental health of children born very preterm. Associations between linguistic distance and preschool children's mental health (SDQ total score) for selected language groups in the German Neonatal Network study sample are presented.

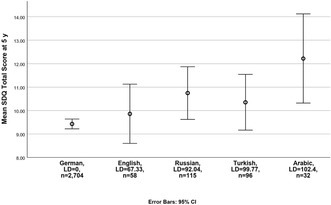

**This original article is commented by Müller et al. on pages** 557–558 of this issue.

Plain language summary: https://onlinelibrary.wiley.com/doi/10.1111/dmcn.16165

**Abbreviations**
BPD: bronchopulmonary dysplasia
GNN: German Neonatal Network
IVH: intraventricular haemorrhage
SDQ: Strengths and Difficulties Questionnaire

**What this paper adds**

- Immigrant children born very preterm face systemic inequalities such as language barriers.
- Language barriers can be operationalized as a continuous linguistic distance score.
- Linguistic distance measures differences between first language and countries' official languages.
- Linguistic distance is associated with behaviour and emotions of children born very preterm.

Globally, more than 13 million infants (>10% of all births) are born preterm (<37 weeks' gestational age) every year.^1^ With improvements in neonatal care, the survival rate of infants at highest risk for developmental impairment, those born very preterm (<32 weeks gestational age) continues to increase. In Germany, 1% to 2% of all infants are born very preterm.^1^ However, increased survival has not resulted in equally improved life chances and quality of life.^2^ Children born very preterm today are still growing up with a highly increased risk for a cluster of neurodevelopmental and mental health problems. These include persistent difficulties with attention, emotions, social behaviour, cognition, language, sensory, and motor skills.^3^ The early difficulties of children born very preterm cascade into lifelong increased risks for mental health problems and academic underachievement.^4^ In addition, studies unequivocally show that developmental outcomes of children born very preterm vary according to sociocultural contexts such as family education, income, and ethnicity.^4^, ^5^ However, research has neglected the role of immigrant background and language barriers for the development of children born very preterm. The few studies that do exist have focused on associations of very preterm birth and immigrant background with short‐term outcomes in infancy.^6^ One study from the Netherlands found that multilingualism was associated with low cognitive and verbal outcomes of children born very preterm at 2 years and 5 years of age.^7^ In a French very preterm cohort, non‐European‐born immigrant status was associated with increased risk for behavioural, psychosocial, and multidomain impairments at age 5 years 6 months.^8^

In 2020, there were 281 million immigrants worldwide, an increase of 62% from 2000.^9^ Europe accounts for the largest rise in immigrants;^9^ social, educational, and health care systems are struggling to cater to immigrants' needs. In the health sector, systemic language barriers pose serious challenges to equity and equality of care.^10^ In Germany, almost 42% of children live in families with at least one immigrant parent,^11^ and they more often struggle with socioeconomic inequalities than children without immigrant parents (hereafter referred to as ‘German children’).^12^ At the same time, immigrant children are at higher risk for behavioural and emotional problems than their German peers.^13^ However, such findings are confounded by intersectionality of inequalities such as low education that pose barriers to immigrant families' access to health care.^14^, ^15^

As a result of migration, linguistic diversity is also growing exponentially worldwide. Immigrants' proficiency in a country's official majority language correlates with educational attainment,^16^ health care utilization,^17^, ^18^ and overall health.^19^ Speakers of other languages than their host countries' official majority languages experience different levels of barriers, depending on the degree of similarity with their first language. For example, some languages are relatively mutually intelligible within their language families, such as Danish, Swedish, and Norwegian,^20^ making them easily attainable for their speakers. Accordingly, some immigrant groups have advantages over others, because of linguistic similarities of their first language to the host society's official majority language(s). To operationalize such variations in language barriers, we assess the role of linguistic distance between children's first language and the official language of the host country. High linguistic distance (i.e. greater distance between two languages) has been associated with poor health^21^ and low educational attainment.^22^ In Germany, linguistic distance may indicate the level of similarities and differences between Turkish, Arabic, Italian, English, and German. Children whose first language is Turkish, for example, need to overcome a higher linguistic distance to the majority language German (linguistic distance = 99.77 points) than children whose first language is Italian in Germany (linguistic distance = 86.30 points) (Figure 1).

**FIGURE 1:**
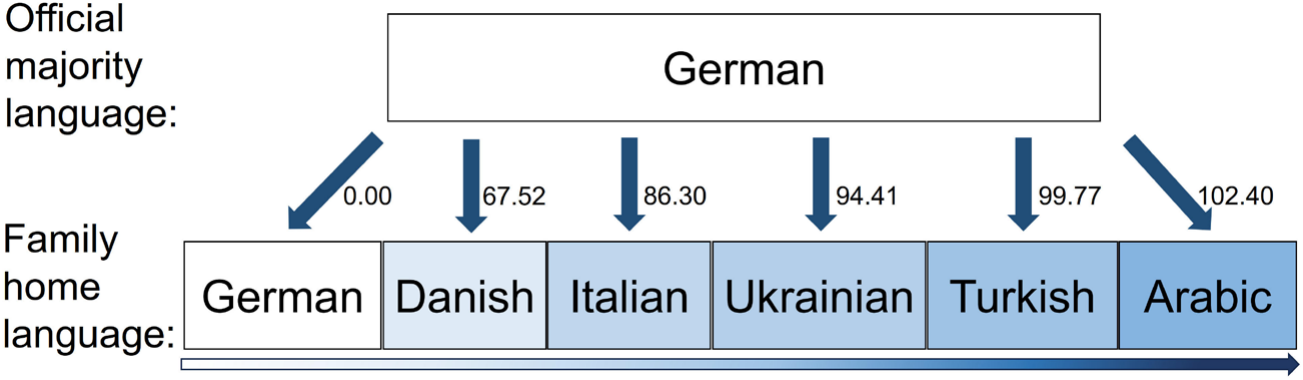
Linguistic distance variable scores between German and five selected immigrant languages.

Very preterm birth, immigrant status, and language barriers in the form of linguistic distance may all be independently associated with high risk for behavioural and emotional problems in childhood. Language barriers and intersectional inequalities of immigrant children born very preterm have been neglected by research. Accordingly, the aim of this study is to assess whether behavioural and emotional problems of 5‐ to 6‐year‐old preschool children born very preterm across Germany are associated with immigrant background and linguistic distance of their first language to the host country's official language, German.

We tested the following hypotheses: (1) immigrant status and linguistic distance of children's first language to German are both associated with behavioural and emotional problems of children born very preterm; (2) linguistic distance is independently associated with behavioural and emotional problems of children born very preterm, after adjusting for the effects of immigrant status, maternal level of education, maternal age, infant sex, gestational age, multiple birth status, intraventricular haemorrhage (IVH), and bronchopulmonary dysplasia (BPD), and accounting for the nestedness of data within birth hospitals.

## METHOD

Data are from the national multicentre observational German Neonatal Network (GNN) cohort study, including all births between 22 + 0 weeks and 31 + 6 weeks gestational age from 1st April 2009 until 31st December 2016 in 68 German neonatal intensive care units.^23^

### Procedures

After obtaining written informed consent from parents or legal guardians, predefined data on general neonatal characteristics, antenatal and postnatal treatment, and outcome were recorded for each patient. After discharge, data sheets were sent to the study centre (University of Lübeck). A physician or study nurse from the study centre with expertise in neonatology monitored the data quality of completed record files through annual on‐site visits. At the age of 5 to 6 years, before children entered formal schooling in Germany, families were invited to the GNN follow‐up investigation at each respective study site. Children were examined by the GNN study team (physician trained in neonatology and two study nurses) regarding their motor and cognitive development and parents answered questions about mental health. Because of funding constraints, the study design at follow‐up included an assessment of 30% of the original GNN study participants. Details of the recruiting process have been published; the follow‐up sample included higher rates of multiples and German ancestry but no differences with regard to gestational age, birthweight, or neonatal complications.^23^ A total of 3220 children were followed up and had complete data; 647 of these had an immigrant background.

### Ethics

All study parts were approved by the University of Lübeck Ethical Committee and the committees of the participating centres (vote no. 08–022). Written informed consent was obtained from parents. All methods were carried out in accordance with relevant guidelines and regulations, specifically: the Declaration of Helsinki, the current revision of the International Council for Harmonisation of Technical Requirements for Pharmaceuticals for Human Use Topic E6, the Guidelines for Good Clinical Practice, the Guidelines of the Council for International Organization of Medical Sciences, and the World Health Organization's ‘Proposed International Guidelines for Biomedical Research Involving Human Subjects’.

### Instruments

#### Children's behavioural and emotional problems

Behavioural and socio‐emotional difficulties were assessed using the parent‐reported Strengths and Difficulties Questionnaire (SDQ). The SDQ is a cross‐culturally valid, short screening instrument containing 25 items that load on five subscales measuring emotional problems, conduct problems, hyperactivity‐inattention, peer relationship problems, and prosocial behaviour.^24^ Each item is scored on a 3‐point Likert‐type scale (0 = not true, 1 = somewhat true, 2 = certainly true). A SDQ total score is obtained by summing the scores of the four problem scales (range 0–40, with some items reverse‐coded).

#### Immigrant status and linguistic distance

Immigrant status was operationalized as a binary variable based on children's mothers' country of birth (born in Germany vs foreign‐born). Children's first language (i.e. the languages spoken in the home) were assessed via free‐form fields in parent questionnaires at age 5 to 6 years. This information was used to calculate the average linguistic distance to German as one continuous variable (range 0–103.73; see Appendix S1 for details) for each child according to the Automated Similarity Judgement Program.^25^ The linguistic distance calculated by the Automated Similarity Judgement Program is based on 40 universally important, culturally independent everyday words and based on the normalized Levenshtein distance (i.e. the number of changes, including deletions, insertions, or substitutions, required to transform the phonetic representation of a word from one language to another). For example, fish (English) to Fisch (German) has a Levenshtein distance of 2, whereas fish (English) to balık (Turkish) has a distance of 5. For a detailed discussion of the statistical procedures for calculating the Automated Similarity Judgement Program linguistic distance score see Wichmann et al.^26^

#### Social, biological, and perinatal clinical variables

Information on mothers' education was collected via questionnaires and binary coded into having obtained a high‐school degree (German Abitur) versus not having a high‐school degree. Information on mothers' age at birth (years), child gestational age (weeks), biological sex (female vs male), multiple birth status (singleton vs multiple), neonatal complications such as BPD (defined as any supplemental oxygen or respiratory support at a gestational age of 36 weeks), and higher grade (>II) IVH was collected from medical records.

#### Intelligence

At 5 to 6 years, children's IQ total scores were assessed with the standardized Wechsler Preschool and Primary Scale of Intelligence, Third Edition, German adaption and standardization.

#### Analysis strategy

Participants with complete data at age 5 to 6 years were included in the main analyses (i.e. complete case analysis). Descriptive statistics and mixed‐effects linear regressions were carried out in Stata version 17.0 (StatCorp, College Station, TX, USA). First, to separately test univariate associations of linguistic distance and immigrant status of children born very preterm with their behavioural and emotional problems we ran two two‐level models (i.e. level 1: individuals; level 2: birth hospitals), one including a fixed effect of children's linguistic distances from their first language to German, the other including a fixed effect of immigrant status. Next, we entered both predictors into one model to adjust their effects for each other. To adjust for a priori known confounders, we then added fixed effects of maternal level of education, maternal age, infant sex, gestational age at birth, multiple birth status, IVH, BPD, and child age at assessment.

## RESULTS

Table 1 outlines descriptive information for the cohort of children followed up at 5 to 6 years comparing German (*n* = 2591) versus foreign‐born mothers (*n* = 629). On average, foreign‐born mothers were older and less likely to have a high‐school degree than German mothers. As expected, most of the children born to German mothers spoke German as their first language (median linguistic distance = 0.00), while there was large variation in linguistic distance scores among children of foreign‐born mothers. Among the diverse language groups represented in the sample, the largest were Russian, Turkish, English, and Arabic. It is important to note that 6.9% of children with German mothers had other first languages than German, indicating substantial linguistic diversity because of immigrant heritage. Children of foreign‐born mothers were older and they had lower IQ scores than children of German mothers.

**TABLE 1:** Descriptive characteristics of the GNN sample assessed at 5 years by German (*n* = 2591) vs foreign‐born mothers (*n* = 629).

	Mother's country of birth		
	German	Foreign	*p*
Infant gestational age (mean [SD], weeks; *n* = 3220)	28.14 (2.39)	28.04 (2.60)	0.352
Mother's age (mean [SD], years:months; *n* = 3208)	31:6 (5:4)	32:4 (5:6)	< 0.001
Mother's educational level (*n* [%]; *n* = 3112)			< 0.001
High‐school degree (German Abitur)	1280 (50.9)	233 (39.0)	
No high‐school degree	1235 (49.1)	364 (61.0)	
Type of pregnancy (*n* [%]; *n* = 3220)			0.057
Singleton	1549 (59.8)	402 (63.9)	
Multiple	1042 (40.2)	227 (36.1)	
Child sex (*n* [%]; *n* = 3220)			0.124
Female	1246 (48.1)	324 (51.5)	
Male	1345 (51.9)	305 (48.5)	
IVH (*n* [%]; *n* = 3220)			0.779
Yes	445 (17.2)	111 (17.6)	
No	2146 (82.8)	518 (82.4)	
BPD (*n* [%]; *n* = 3219)			0.612
Yes	489 (18.9)	113 (18.0)	
No	2102 (81.1)	515 (82.0)	
Linguistic distance to German (mean [SD]; median; *n* = 3220)	6.06 (22.57); 0.00	49.49 (46.82); 80.66	< 0.001
Child age at assessment (mean [SD], days; *n* = 3220)	2030.93 (156.83)	2045.56 (158.78)	0.036
SDQ total score at 5–6 years (mean [SD]; *n* = 3220)	9.52 (5.51)	9.89 (5.63)	0.132
IQ score at 5–6 years (mean [SD]; *n* = 2732)	100.32 (12.63)	95.22 (14.33)	< 0.001

Abbreviations: BPD, bronchopulmonary dysplasia; IVH, higher grade (> II) intraventricular haemorrhage; SDQ, Strengths and Difficulties Questionnaire.

**FIGURE 2:**
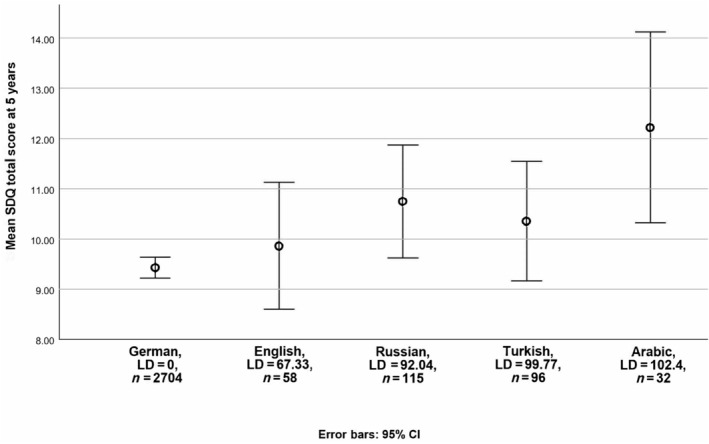
Unadjusted effect of linguistic distance (LD) on children's behavioural and emotional problems (Strengths and Difficulties Questionnaire [SDQ] total score) at 5 to 6 years for selected language groups in the sample. Abbreviation: CI, confidence interval.

Unadjusted models showed that higher linguistic distance was associated with higher SDQ total scores (Table 2, Model 1). Mothers' foreign country of birth (i.e. immigrant status) was not associated with higher SDQ total scores (coefficient = 0.440; 95% confidence interval [CI] = −0.010, 0.890). Linguistic distance remained significant (coefficient = 0.01; 95% CI = 0.01, 0.02) when both linguistic distance and immigrant status were included simultaneously in one model (Model 2). Random effects indicated substantial variation in the association of linguistic distance with SDQ total scores according to birth hospitals (coefficient = 0.63; 95% CI = 0.39, 1.00). Finally, in Model 3 linguistic distance from children's first language to German was independently associated with SDQ total scores (coefficient = 0.01; 95% CI = 0.00, 0.02) after adjusting for confounders (i.e. maternal level of education, maternal age, infant sex, gestational age at birth, multiple birth status, IVH, BPD, and age at assessment). As before, random effects indicated variation in the association between children's linguistic distance and SDQ total scores according to birth hospitals. In this model, if all other factors were held stable, a 10‐point higher linguistic distance would result in a 0.10‐point higher SDQ total score. Accordingly, for example, the SDQ total problem score would be on average 1 full point higher among Turkish‐speaking immigrant children living in Germany (linguistic distance = 99.77, *n* = 96) compared with German children. Figure 2 shows unadjusted effects for selected language groups in the sample.

**TABLE 2:** Multilevel linear mixed‐effects models showing associations of the linguistic distance of children born very preterm with SDQ total scores at age 5 years.

	SDQ total score		
Dependent variable	Model 1 (Participants = 3220; birth hospitals = 50; participants per hospital min. = 5, max. = 150, mean = 64)	Model 2 (Participants = 3220; birth hospitals = 50; participants per hospital min. = 5, max. = 150, mean = 64)	Model 3 (Participants = 3099; birth hospitals = 50; participants per hospital min. = 5, max. = 143, mean = 62)
**Fixed effects, coefficient (95% confidence interval)**			
Linguistic distance	**0.012 (0.006, 0.017)** ^a^	**0.012 (0.006, 0.019)** ^a^	**0.008 (0.002, 0.015)** ^b^
Mother foreign country of birth		−0.074 (−0.637, 0.489)	−0.008 (−0.561, 0.545)
Mother's education (high vs low)			−1.272 (−1.650, −0.893)^a^
Mother's age (years)			−0.101 (−0.136, −0.065)^a^
Gestational age (weeks)			−0.267 (−0.352, −0.181)^a^
Biological sex (female vs male)			−0.933 (−1.304, −0.561)^a^
Multiple birth			−1.120 (−1.501, −0.738)^a^
IVH			1.021 (0.510, 1.533)^a^
BPD			1.703 (1.185, 2.221)^a^
Age at assessment (days)			0.001 (0.000, 0.003)^b^
constant	9.423 (9.142, 9.704)^a^	9.505 (8.822, 10.189)^a^	20.450 (16.889, 24.010)^a^
**Random effects by birth hospital**			
SD (constant)	0.627 (0.395, 0.995)	0.625 (0.392, 0.995)	0.373 (0.150, 0.928)
**Log‐pseudolikelihood**	−10 067.838	−10 067.805	−9 534.279

Model 1 = unadjusted; Model 2 = linguistic distance and immigrant status adjusted for each other; Model 3 = fully adjusted, including linguistic distance, immigrant status, maternal level of education, maternal age, infant gestational age, sex, multiple birth status, IVH, and BPD; two‐tailed significance level for fixed effects.

Significant effects of interest are marked in bold.

Abbreviations: BPD, bronchopulmonary dysplasia; IVH, higher grade (>II) intraventricular haemorrhage; min., minimum; max., maximum; SDQ, Strengths and Difficulties Questionnaire.

^a^ *p* ≤ 0.001.

^b^ *p* < 0.05.

Analyses were repeated within the subgroup of children with foreign‐born mothers to assess the specific size of the association between linguistic distance and the SDQ total scores of immigrant children born very preterm (Table 3). As in the full sample, the fixed effect of children's linguistic distances to German on SDQ total scores remained stable after adjusting for confounders (coefficient = 0.01; 95% CI = 0.00, 0.02).

**TABLE 3:** Multilevel linear mixed‐effects models showing associations of linguistic distance of immigrant children born very preterm with SDQ total scores at age 5 years.

	SDQ total score	
Dependent variable	Model 1 (Participants = 629; birth hospitals = 49; participants per hospital min. = 1, max. = 45, mean = 13)	Model 2 (Participants = 593; birth hospitals = 49; participants per hospital min. = 1, max. = 42, mean = 12)
**Fixed effects, coefficient (95% confidence interval)**		
Linguistic difference	**0.012 (0.003, 0.022)** ^a^	**0.011 (0.001, 0.020)** ^a^
Mother's education (high vs low)		−0.694 (−1.594, 0.206)
Mother's age (years)		−0.109 (−0.189, −0.029)^b^
Gestational age (weeks)		−0.120 (−0.315, 0.076)
Biological sex (female vs male)		−0.313 (−1.186, 0.560)
Multiple birth		−1.222 (−2.152, −0.293)^b^
IVH		0.856 (−0.327, 2.040)
BPD		2.382 (1.132, 3.631c)^c^
Age at assessment (days)		0.003 (0.000, 0.006)^a^
constant		11.470 (3.042, 19.897)^c^
**Random effects by birth hospital**		
SD (constant)	0.237 (0.000, 283.520)	0.215 (0.000, 1110.179)
**Log‐pseudolikelihood**	−1976.085	−1838.689

Model 1 = unadjusted; Model 2 = fully adjusted, including linguistic distance, maternal level of education, maternal age, infant gestational age, sex, multiple birth status, IVH, and BPD; two‐tailed significance level for fixed effects.

Significant effects of interest are marked in bold.

Abbreviations: BPD, bronchopulmonary dysplasia; IVH, higher grade (>II) intraventricular haemorrhage; max., maximum; min., minimum; SDQ, Strengths and Difficulties Questionnaire.

^a^ *p* < 0.05.

^b^ *p* ≤ 0.01.

^c^ *p* ≤ 0.001.

We carried out a sensitivity analysis, additionally including a fixed effect of children's IQ scores as confounder in the fully adjusted mixed‐effects model. Because of missing IQ data for some children the sample size was lower (*n* = 2657 for the fully adjusted model). While IQ was associated with children's SDQ scores (coefficient = −0.07; 95% CI = −0.09, −0.06), the effect of linguistic distance was attenuated (coefficient = 0.01; 95% CI = −0.00, 0.01).

## DISCUSSION

This study confirms an association of linguistic distance between the first language of children born very preterm and their host country's official majority language with higher behavioural and emotional problems at 5 years of age.^27^ Our findings from the large German‐wide longitudinal GNN cohort study of children born very preterm suggest that there is no direct association of immigrant status with behavioural and emotional problems, but that instead language barriers are an explanatory factor. Importantly, the contribution of linguistic distance to explaining the behaviour of children born very preterm remained stable even after adjusting for social, biological, and perinatal clinical factors such as IVH and BPD. This points to a fundamental role of language barriers for the development of immigrant children born very preterm.

It is important to note that the size of the fixed effect of linguistic distance (i.e. coefficient = 0.01; CI = 0.00, 0.02) in our models may seem small in comparison to other variables' effects. However, the mixed‐model coefficients represent the change in the mean response (SDQ total score) associated with a 1‐unit change in that term. Accordingly, for example, if all other factors in the model would be held stable, the SDQ total problem score would be on average more than 1 full point higher among Arabic‐speaking immigrant children living in Germany compared with non‐immigrant German children. Considering the SD of mean SDQ total scores, one may argue whether one‐fifth SD difference is meaningful. However, response differences on one Likert‐scaled SDQ item may indicate meaningful subclinical differences in children's everyday behaviours. In reality, children's language barriers are associated with other risks such as low maternal education or low IQ, which for an individual child present clusters of multiple risks. While researchers must rely on clearly defined hypothesis testing, it is also important to raise awareness for this intersectionality of risks. The small but significant association between linguistic distance and SDQ total scores provides novel pointers for specific prevention studies taking language barriers into account.

Immigrant status alone, operationalized as a binary category in German versus foreign‐born mothers, was not associated with children's behaviour and emotions. This supports assessing systemic variables such as language barriers when studying immigrant children's development.^17^, ^18^, ^21^ There are wide variations between immigrants' experiences depending on a range of intersectional factors including their heritage languages, countries of origin, reasons for migration, legal status, ethnicity, educational qualifications, and economic resources.^14^ The traditionally used oversimplification of the variable ‘immigrant’ is often masking these heterogeneous conditions and intersectional inequalities shaping immigrants' lived experiences. The operationalization of language barriers via linguistic distance helps explain important aspects of this complex situation.^22^ Researchers from other geographic regions may consider implementing linguistic distance as a screening tool to assess whether these findings from Germany are replicated. Based on the standardized operationalization of the linguistic distance score variable, we believe that the current findings are not specific for German or Germany, but that language barriers in the form of linguistic distance are a universal, global phenomenon.

As expected, social, biological, and perinatal clinical factors made important contributions to children's behavioural and socio‐emotional problems. There is substantial overlap among these vulnerabilities, creating additive risks for certain individuals. In particular when working with immigrant groups, it is important to stress the contribution of the mother's level of education to their children's behavioural and emotional problems.^13^ Foreign‐born mothers in the current sample were less likely than German mothers to have completed high school. If all other factors in the model were held stable, children of mothers with a high‐school degree (i.e. German Abitur) would have on average 1.28 lower points on the SDQ total score compared with children of mothers who did not complete high school. The sensitivity analysis including a fixed effect of children's IQ scores as additional confounder in a subsample showed that IQ was associated with children's SDQ scores while the effect of linguistic distance was attenuated. This points to the well‐known association between systemic inequalities, language barriers, biases in cognitive assessment, and perceived behaviour problems children from immigrant families are facing.^15^

The random effects included in our nested models showed small but stable variations in the association between children's linguistic distance and SDQ total scores according to birth hospitals. Considering regional and organizational differences between hospitals' patient populations (e.g. percentage of immigrants), catchment areas (e.g. rural vs urban, socioeconomic distribution), and individual treatment experiences, this is not surprising. Nevertheless, the current data suggest that treatment disparities between hospitals in Germany may not be characterized by as severe inequalities as reported by US‐based studies.^28^

### Strengths and limitations

The main strength of this multicentre, prospective cohort study is the large sample size of children born very preterm across Germany. The operationalization of language barriers in the form of linguistic distance allowed us to break up the oversimplified categorization of participants into ‘native vs foreign‐born’. At the same time, the continuous linguistic distance score provides an elegant solution to assess variations in language barriers. Our operationalization allowed for more than one first language to be considered, as is often the lived reality in families' lives,^22^ and we calculated the average linguistic distance of the reported non‐German languages. German was very likely a part of every child's life through preschool and other out‐of‐home contexts, but research on linguistic distance has shown that language barriers remain even with good German skills. Children's actual language proficiency in German or other languages was not tested as part of this study. Rather, we aim to provide proof‐of‐concept that a simple and easy‐to‐implement screening variable, such as the linguistic distance score based on self‐reported home language, is meaningfully associated with the development of children born very preterm. In other words, we understand linguistic distance as a proxy variable to assess potential language barriers in health care and education.^21^, ^22^ We encourage future research to use this tool to replicate our findings in other populations and contexts. Moreover, practitioners and policymakers may consider broad implementation in conjunction with migrant friendly assessments to facilitate immigrant‐sensitive, equitable health care provision promoting the human rights of children with complex chronic conditions.^29^

This study also has weaknesses. Despite its continuous scoring, linguistic distance was not normally distributed across the population. However, its effect remained stable throughout all models and overall fit values indicated robust findings. The binary variable ‘immigrant vs German’ was operationalized based on mothers' country of birth, since fathers' information was only available for a subgroup. As a result, we may have misclassified children with immigrant fathers as ‘German’. Fathers play an important role in their children's development. Unfortunately, it was beyond the scope of this manuscript to include their information. Because of funding constraints, the GNN sample participating in the 5‐ to 6‐year follow‐up only included 30% of the originally enrolled infants. Unfortunately, we cannot assess how participants differ from the original sample, because immigrant status, language use, and maternal education were only assessed at the 5‐ to 6‐year follow‐up. However, data of the GNN correspond well with national German data.^30^ At birth, national ancestry was recorded, operationalized as ethnic heritage based on categories of world regions. For this variable, the percentage of participants with ‘German ancestry’ increased from 71% at birth to 77% at 5 to 6 years.^23^ Even though a different subgroup of the GNN was analysed for the current study, this gives an indication that more immigrants than German families may have been lost to follow‐up.

Moreover, the SDQ and all other assessments were administered to participating parents in German only, not in immigrant families' first languages. This has likely created participation bias (due to a higher proportion of foreign‐born mothers dropping out whose German language skills were not sufficient) as well as response bias (due to misunderstandings of instructions and item content). Likewise, standardized IQ assessments were only administered in German, irrespective of children's language backgrounds. IQ has a complex relationship with developmental outcomes across different risk groups. Our sensitivity analysis on a subsample showed that IQ was associated with children's SDQ scores, while the effect of linguistic distance was attenuated. The purely German test administration has likely created assessment biases and inequities, underestimating immigrant children's IQs. We therefore caution readers to carefully interpret this association. Future studies should ensure that all assessments are administered in participants' first language, and to select standardized instruments that are as culturally fair as possible. Participating foreign‐born mothers' educational qualifications were only assessed according to the German educational system, so that mothers who had obtained Abitur‐equivalent degrees abroad were coded into the wrong category. Although the data show an effect of mothers' education on children's behavioural and emotional problems, we suspect it has been underestimated. Future studies should pay better attention to assess immigrants' educational qualifications in ways that allow globally comparable operationalization, for instance with the International Standard Classification of Education coding system. Based on the current findings, it is not clear whether the reported effect of linguistic distance is also observed in other developmental domains that shape the preterm phenotype (e.g. cognition or motor skills). Other factors such as maternal mental health and social support may be associated with children's SDQ scores but were not measured in this study. Finally, the limited available literature points to a multiplicative developmental risk of language barriers and preterm birth.^7^, ^27^ We could not test this hypothesis with the current sample as all children were born very preterm.

### Conclusion

A higher linguistic distance between the first language of children born very preterm and German is associated with higher behavioural and emotional problems at 5 to 6 years of age. Language barriers play an important role for the development of immigrant children born very preterm. Researchers, practitioners, and policymakers may consider implementation of screening for linguistic distance as part of regular follow‐up after very preterm birth.

## Supporting information


**Appendix S1:** List of languages spoken by children in the GNN sample including reported frequency and linguistic distance to German by German vs foreign‐born mothers

## Data Availability

The data that support the findings of this study are available in aggregated form upon reasonable request from the corresponding author. The data are not publicly available due to privacy and ethical restrictions.
