# Supplementary material for: Language barriers and mental health problems of preschool children born very preterm in Germany
Source: Dev Med Child Neurol. 2024 Oct 21;67(5):600–8. doi: 10.1111/dmcn.16132 (PMC11965972; doi:10.1111/dmcn.16132)
Supplement: Supplementary file 1 — Appendix S1: List of languages spoken by children in the GNN sample including reported frequency and linguistic distance to German by German vs foreign‐born mothers [file DMCN-67-600-s001.docx]

**Appendix 1**

**List of languages spoken by children in the GNN sample including reported frequency (*n)* and linguistic distance (LD score) to German by German vs. foreign-born mothers (*N*=3,220)**

| **Languages** | **German mothers**  ***n*** | **Foreign-born mothers**  ***n*** | **LD score** |
| --- | --- | --- | --- |
| German | 2,412 | 292 | 0.00 |
| Dutch | 4 | 0 | 48.83 |
| English | 42 | 16 | 67.33 |
| Danish | 4 | 2 | 67.52 |
| Swedish | 0 | 1 | 69.79 |
| Dutch & Portuguese^1^ | 0 | 2 | 71.18 |
| English & Russian^1^ | 0 | 1 | 79.69 |
| English & Spanish^1^ | 2 | 0 | 80.16 |
| Hindi & English^1^ | 0 | 2 | 80.66 |
| English & Twi^1^ | 1 | 2 | 83.40 |
| English & Turkish^1^ | 1 | 0 | 83.55 |
| Filipino | 0 | 2 | 83.70 |
| Italian | 8 | 3 | 86.30 |
| Romanian | 1 | 3 | 88.17 |
| Macedonian | 0 | 5 | 88.94 |
| Spanish & Italian | 1 | 0 | 89.55 |
| Portuguese & Italian^1^ | 0 | 1 | 89.92 |
| Greek & Italian^1^ | 1 | 0 | 90.84 |
| Serbian, Croatian^2^ | 3 | 14 | 91.56 |
| Russian & Serbian^1^ | 0 | 1 | 91.80 |
| Russian | 2 | 113 | 92.04 |
| Bulgarian | 0 | 5 | 92.10 |
| Dari Persian | 1 | 4 | 92.89 |
| Spanish | 14 | 3 | 92.98 |
| Portuguese | 7 | 9 | 93.53 |
| Hindi | 0 | 1 | 94.00 |
| Ukrainian | 1 | 4 | 94.41 |
| Greek | 8 | 4 | 95.37 |
| Albanian | 0 | 20 | 95.68 |
| French | 5 | 6 | 95.73 |
| French & Malagasy^1^ | 0 | 3 | 95.78 |
| Kurdish | 1 | 11 | 96.71 |
| Moroccan | 3 | 4 | 96.74 |
| Pashto | 1 | 3 | 96.78 |
| Polish & Turkish^1^ | 1 | 0 | 98.14 |
| Turkish & Kurdish^1^ | 0 | 2 | 98.24 |
| Hungarian | 0 | 8 | 98.33 |
| Greek & Arabic^1^ | 2 | 0 | 98.89 |
| Azerbaijani & Arabic^1^ | 0 | 1 | 99.11 |
| Twi | 0 | 1 | 99.50 |
| Tamil | 0 | 6 | 99.67 |
| French & Lingala^1^ | 0 | 1 | 99.76 |
| Turkish | 47 | 49 | 99.77 |
| Tamazight | 0 | 1 | 100.48 |
| Lebanese | 0 | 1 | 101.32 |
| Chinese | 0 | 4 | 102.35 |
| Arabic | 15 | 17 | 102.40 |
| Lingala | 1 | 1 | 103.73 |
| **Total *N*** | 2,591 | 629 |  |

^1^ if parents had indicated that their children had multiple L1s, these languages’ linguistic distance to German was averaged; ^2^ according to the Automated Similarity Judgement Program (ASJP) (1), these languages have the same linguistic distance to German

**Reference**

1. Wichmann S, Brown CH, Holman EW. The ASJP Database. Jena: Max Planck Institute for the Science of Human History; 2020.
